# Is It a Fact?

**Published:** 1857-04

**Authors:** 


### IS IT A FACT?

A RELIGIOUS newspaper in Philadelphia announces in its column of *General News*, "Not more necessary for the preservation of life on the battle field is a complete armor, than is a bottle of * * * pain killer to those who are suffering from acute bodily pain."

The above is neither new nor true, and that it should be placed among items of news, when it is paid for as an advertisement, is to our mind not good morals in any paper, secular or religious.

